# Progesterone metabolites regulate induction, growth, and suppression of estrogen- and progesterone receptor-negative human breast cell tumors

**DOI:** 10.1186/bcr3422

**Published:** 2013-05-11

**Authors:** John P Wiebe, Guihua Zhang, Ian Welch, Heather-Anne T Cadieux-Pitre

**Affiliations:** 1 Department of Biology, The University of Western Ontario, London, Ontario, N6A5B7 Canada; 2 Department of Anatomy & Cell Biology, Schulich School of Medicine & Dentistry, The University of Western Ontario, London, Ontario, N6A 5C1 Canada; 3 Department of Animal Care & Veterinary Services and Department of Physiology and Pharmacology, Medical Sciences Building, The University of Western Ontario, London, Ontario, N6A 5C1 Canada; 4 Department of Animal Care & Veterinary Services, Medical Sciences Building, The University of Western Ontario, London, Ontario, N6A 5C1 Canada

**Keywords:** Breast cancer, ER/PR-negative breast cancers, hormonal control, microenvironment, progesterone metabolites, 5α-dihydroprogesterone, 3α-dihydroprogesterone, tumorigenesis, tumor promoter and suppressor hormones, biomarkers, normalcy

## Abstract

**Introduction:**

Of the nearly 1.4 million new cases of breast cancer diagnosed each year, a large proportion is characterized as hormone receptor negative, lacking estrogen receptors (ER) and/or progesterone receptors (PR). Patients with receptor-negative tumors do not respond to current steroid hormone-based therapies and generally have significantly higher risk of recurrence and mortality compared with patients with tumors that are ER- and/or PR-positive. Previous *in vitro *studies had shown that the progesterone metabolites, 5α-dihydroprogesterone (5αP) and 3α-dihydroprogesterone (3αHP), respectively, exhibit procancer and anticancer effects on receptor-negative human breast cell lines. Here *in vivo *studies were conducted to investigate the ability of 5αP and 3αHP to control initiation, growth, and regression of ER/PR-negative human breast cell tumors.

**Methods:**

ER/PR-negative human breast cells (MDA-MB-231) were implanted into mammary fat pads of immunosuppressed mice, and the effects of 5αP and 3αHP treatments on tumor initiation, growth, suppression/regression, and histopathology were assessed in five separate experiments. Specific radioimmunoassays and gas chromatography-mass spectrometry were used to measure 5αP, 3αHP, and progesterone in mouse serum and tumors.

**Results:**

Onset and growth of ER/PR-negative human breast cell tumors were significantly stimulated by 5αP and inhibited by 3αHP. When both hormones were applied simultaneously, the stimulatory effects of 5αP were abrogated by the inhibitory effects of 3αHP and vice versa. Treatment with 3αHP subsequent to 5αP-induced tumor initiation resulted in suppression of further tumorigenesis and regression of existing tumors. The levels of 5αP in tumors, regardless of treatment, were about 10-fold higher than the levels of 3αHP, and the 5αP:3αHP ratios were about fivefold higher than in serum, indicating significant changes in endogenous synthesis of these hormones in tumorous breast tissues.

**Conclusions:**

The studies showed that estrogen/progesterone-insensitive breast tumors are sensitive to, and controlled by, the progesterone metabolites 5αP and 3αHP. Tumorigenesis of ER/PR-negative breast cells is significantly enhanced by 5αP and suppressed by 3αHP, the outcome depending on the relative concentrations of these two hormones in the microenvironment in the breast regions. The findings show that the production of 5αP greatly exceeds that of 3αHP in ER/PR-negative tumors and that treatment with 3αHP can effectively block tumorigenesis and cause existing tumors to regress. The results provide the first hormonal theory to explain tumorigenesis of ER/PR-negative breast tissues and support the hypothesis that a high 3αHP-to-5αP concentration ratio in the microenvironment may foster normalcy in noncancerous breast regions. The findings suggest new diagnostics based on the relative levels of these hormones and new approaches to prevention and treatment of breast cancers based on regulating the levels and action mechanisms of anti- and pro-cancer progesterone metabolites.

## Introduction

Breast cancer is the most frequently diagnosed cancer and the leading cause of cancer death in women worldwide, with nearly 1.4 million new cases annually [1]. Progesterone and estrogens have long been linked to breast cancer [2,3], and current understanding of the effective actions of these hormones implies the presence of receptors (ER and PR) in the target cells [4,5]. However, a large proportion (about 30% to 60%) of breast tumors are ER and/or PR negative [4,6-8], and about 90% of normal proliferating breast epithelial cells are receptor negative [9]. Patients with receptor-negative tumors generally show lack of response to adjuvant hormone therapy and have significantly higher risk of mortality compared with patients with tumors that are ER and/or PR positive [10-14]. Overall, this means that for receptor-negative breast cancers, current explanations based on estrogen and progesterone actions and receptors are inadequate, and the related hormone-based therapies are ineffective. Here evidence is presented that the progesterone metabolites, 5α-pregnane-3,20-dione (5α-dihydroprogesterone; 5αP) and 4-pregnen-3α-ol-20-one (3α-dihydroprogesterone; 3αHP), can regulate ER/PR-negative breast cell tumor formation and growth as well as tumor regression and maintenance of normalcy.

**Figure 1:**
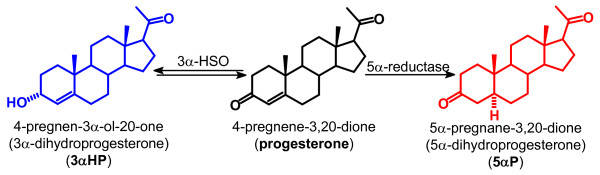
**Conversion of progesterone to 3α-dihydroprogesterone (3αHP) and 5α-dihydroprogesterone (5αP)**. *In vitro *studies have shown that both ER/PR-positive and -negative human breast tissues and cell lines are able to convert progesterone to 3αHP and 5αP by the actions of 3α-hydroxysteroid oxidoreductase (3α-HSO) and 5α-reductase, respectively.

Our previous *in vitro *studies had shown that breast tissues and cell lines readily convert progesterone to 5α-pregnanes, such as 5αP, and delta-4-pregnenes, such as 3αHP (Figure 1), and that tumorous breast tissues [15] and tumorigenic breast cell lines [16] produce higher levels of 5αP and lower levels of 3αHP than do normal breast tissues and nontumorigenic cell lines. The differences in progesterone metabolism between normal and tumorous breasts were observed in all breast tissue samples examined, regardless of the ages of the women, subtypes and grades of carcinomas, and whether the tissues were ER and PR positive and/or negative [15]. The progesterone metabolism studies suggested that increases in 5αP and decreases in 3αHP production accompany the shift toward breast cell neoplasia and tumorigenicity [17]. *In vitro *studies on five different human breast cell lines showed that cell proliferation and detachment are significantly increased by 5αP and decreased by 3αHP [15,18]. The opposing *in vitro *effects of 5αP and 3αHP were observed in all breast cells studied: tumorigenic and nontumorigenic, estrogen-responsive and unresponsive, and ER/PR-positive and -negative cells [18].

The objectives of the current studies were (a) to determine whether the progesterone metabolites, 5αP and 3αHP, have the ability to regulate *in vivo *induction and growth of ER/PR-negative human breast cancer cell tumors in mice, and (b) to determine the relative concentrations of 5αP and 3αHP in serum of tumorous and nontumorous mice and within tumors. The studies provide the first *in vivo *evidence that initiation and growth of ER/PR-negative human breast cell tumors are markedly stimulated by 5αP and suppressed by 3αHP, and that established 5αP-induced tumors can be regressed by treatment with 3αHP. Measurements of their levels indicate that the relative concentrations in the breast microenvironment of the progesterone metabolites determine whether ER/PR-negative cells are stimulated toward neoplasia and tumorigenesis or regulated to maintain a normal state.

## Methods and materials

### Chemicals and reagents

Progesterone, 5αP, cell-culture media, insulin, penicillin, and streptomycin were obtained from Sigma Chemical Co. (Oakville, ON, Canada). 3αHP was obtained from Steraloids (Newport, RI, USA). Serum was purchased from Invitrogen (Burlington, ON, Canada). [1,2,6,7-^3^H]Progesterone and [9,11,12-^3^H]5α-pregnan-3α-ol-20-one were purchased from Perkin-Elmer (Woodbridge, ON, Canada). Other chemicals and solvents were of appropriate analytic grade and were purchased from Sigma Chemical Co., BDH Inc., (Toronto, ON, Canada), VWR (Mississauga, ON, Canada), or Fisher Scientific Ltd. (Toronto, ON, Canada). Ethanol was double (glass) distilled.

### Cells

The human breast cell line MDA-MB-231 was obtained from American Type Culture Collection (ATCC, Manassas, VA, USA), and cells were grown in a 1:1 Ham F12 Medium and Dulbecco Modified Eagle Medium with supplements and 10% calf serum as described [18]. Cells were grown in T-75 flasks (Sarstedt) and were harvested at approximately 80% confluence. Cell-proliferation and -detachment responses to 5αP and 3αHP were tested [15] before harvesting for inoculation into animals, and cell viability was determined with the trypan blue exclusion test. Cells intended for inoculation into mice were harvested, washed, and then suspended in serum-free medium (about 5 × 10^6 ^cells per 100 μl).

### Animals

Severe combined immunodeficiency (SCID) female mice with impaired T- and B-cell lymphocyte development (NOD SCID) were obtained from Charles River Laboratories (Saint-Constant, Quebec, Canada) at 5 to 6 weeks of age and maintained under specific pathogen-free conditions with food and water *ad libitum*. All animal handling and procedures were approved by Western University Institutional Care and Use Committee. After acclimation (8 to 27 days), cells were implanted on day 0. Each mouse was anesthetized with a mixture of isofluorane and oxygen, and about 5 × 10^6 ^cells, suspended in 100 μl cold (0°C to 4°C) serum-free medium, were injected into the right thoracic mammary fat pad through a 5-mm incision at the sternum region, by using a 1.0-ml syringe with 26-gauge needle. The wound was closed in one layer with metal wound clips or with tissue adhesive (3M Vetbond; St. Paul, MN, USA). The surgical, injection, and handling procedures were conducted in approved laminar-flow sterility hoods. At termination (asphyxiation by CO_2_), blood was collected, tumors were excised and weighed, necropsies were conducted, and tissues were fixed in 10% formalin for histopathologic observation (5-μm sections, hematoxylin and eosin). Some tumors were stored in methanol for steroid extraction.

### Treatments

Suspensions of 5αP and 3αHP were prepared under sterile conditions in sterile-filtered vehicle (0.9% NaCl in double-distilled H_2_O, containing 0.1% double-distilled ethanol and 0.05% Tween 80) at 4 to 5 mg/150 μl. The suspensions were stored at 4°C before use and were administered SC (150 μl/injection) by using a 1.0-ml syringe with a 23-gauge needle in the nape of the neck.

### Tumor growth monitoring

The growth of tumors was monitored at regular intervals (weekly at first, and after appearance of palpable tumors, every second day or every day). Tumor volumes were determined from digital caliper measurements of length and width. The formula (length × (width)^2 ^× 0.6) was determined to be a good approximation of tumor volumes (mm^3^) as calculated empirically by water-displacement measurements of various irregular tumorlike shapes and sizes of modeling clay.

### Synthesis of [^3^H]-5αP and 5αP-BSA conjugate

[9,11,12-^3^H]5αP was prepared by oxidation of [9,11,12-^3^H]5α-pregnan-3α-ol-20-one, as described [19]. Purification of [^3^H]-labeled 5αP was by high-pressure liquid chromatography (HPLC; C_18 _column and methanol/water, 3:1) and TLC (Fisherbrand silica gel GF; three runs in hexane:ethyl acetate, 5:2) [19,20]. Preparation of 5α-pregnane-3,20-dione-11α-hemisuccinate-BSA (5αP-BSA conjugate) was by previously described procedures [21,22], and purity of the conjugate was confirmed with HPLC in the solvent system acetonitrile:H_2_O:trifluoroacetic acid (45:55:0.1) by using a Vydac C_4 _column (4.6 × 250 mm) for protein with particle size, 5 μm, and pore diameter, 300 A.

### Synthesis of [^3^H]3αHP

Tritiated 3αHP ([1,2,6,7-^3^H]3αHP) was prepared from freshly TLC-cleaned [1,2,6,7-^3^H]progesterone by using potassium trisamylborohydride (KS-Selectride; Aldrich) as reducing agent, as described [20,23], with some modifications. In brief, [^3^H]progesterone (50 to 100 μCi) was transferred to a dry siliconized reaction tube; the ethanol was evaporated under N_2, _and the tube was dried overnight in a vacuum desiccator over gypsum (Drierite). Dry molecular sieves (3 to 4) were added to the tube under an N_2 _cone, and the tube was sealed with a rubber septum. Dry tetrahydrofuran (THF; 200 μl) was added, and the N_2_-purged tube was cooled to -80°C. The reaction was initiated at -80°C with slow dropwise addition of cold KS-Selectride (100 μl) under an N_2_-purged atmosphere and with gentle agitation. After 1 hour, the reaction was continued in an ice bath (0°C) for another 3 hours and terminated with the addition of 1.0 ml THF and 1.5 ml cold (0°C) 0.1N NaOH. The reaction mixture was extracted 3 times with 5 ml ethyl ether or ether:CH_2_Cl_2 _(5:1) and cleaned by backwashing and C_18 _bonded silica gel columns. The reaction products were separated and purified with TLC and HPLC, as described earlier under Synthesis of [^3^H]-5αP, and [^3^H]3αHP was stored in double-distilled ethanol (purged under N_2_), at -20°C.

### Preparation of antisera

The 3αHP antiserum (lyophilized) was from our stock originally generated in rabbits by using a 3αHP-carboxymethyloxime-BSA conjugate [20]. For the preparation of 5αP and progesterone antiserum, two male SPF New Zealand white rabbits were immunized with 5αP-BSA, and titer was determined. The serum was stored at -80°C. The antiserum from Rabbit 1 showed high specificity for 5αP, and low cross reaction (percentage relative to 5αP at 100%) with progesterone (2.2%), 3αHP (1.3%), estradiol (2.2%), 4-pregnen-20α-ol-3-one (0.9%), and other 5α-pregnanes and testosterone (<0.1%). The serum from Rabbit 2 had lower specificity for 5αP but acceptable specificity for progesterone and was therefore used for the progesterone radioimmunoassay (RIA).

### Steroid extractions from serum and tumor tissues

Sera (100 to 300 μl) from 29 mice from different experiments were extracted 3 times with 2.0 ml ether/chloroform (6:1). The water and organic solvent phases were separated by freezing (-80°C), the combined solvent portions were dried down under a stream of N_2_, and the residue was brought up in 0.5 ml of methanol/CH_2_Cl_2 _(5:1), purged with N_2_, and stored at -20°C until chromatography was performed. Tumors were weighed, cut into pieces, and homogenized in 5-ml methanol by using a Polytron and extracted with methanol and 3 times with ether/CH_2_Cl_2 _(5:1). The combined solvent was evaporated under N_2_, and the samples brought up in methanol/CH_2_Cl_2 _(5:1) were cleaned by solid-phase extraction (C_18_-bonded silica gel columns) [20] by using methanol/CH_2_Cl_2 _(20:1) as eluant at a flow rate of about 0.8 ml/min. The fractions containing the steroids were combined, evaporated under N_2_, and brought up in 0.5 ml methanol/CH_2_Cl_2 _(5:1), purged with N_2_, and stored at -20°C until chromatography.

### Chromatographic separation of progesterone, 5αP, and 3αHP

Thin-layer chromatography (TLC) of serum and tumor extracts was performed on 20 × 20-cm silica gel G(F) TLC plates (250 μm; Fisher Scientific, Pittsburgh, PA, USA). Extracts and standards (5αP, 3αHP, progesterone) were run in separate lanes (2×) in a solvent system consisting of hexane/chloroform/ethyl acetate (60:60:30). The standards were located by UV absorption and exposure to iodine vapors and, on average, were located at Rf of 0.62 (5αP), 0.44 (progesterone), and 0.27 (3αHP). Regions in the sample lanes coinciding with the standards were extracted with ether/chloroform (6:1); the extracts were evaporated under N_2 _and brought up in 0.4-ml double-distilled ethanol.

### Radioimmunoassays

The RIAs for 5αP, 3αHP, and progesterone were basically as described [20], with the generated antisera (see earlier) and scintillation spectrometry (Beckman-Coulter LS 6500 Scintillation Counter). For purposes of comparison, concentrations were standardized to nanograms per milliliter for serum and nanograms per gram for tumors, and it was assumed that these two measures represent a reasonable equivalence.

### Mass spectrometry

For verification of RIA measurements of 5αP, 3αHP, and progesterone, portions of TLC-separated extracts from four tumor tissues were tested with both RIA and GC/MS (Hewlett-Packard GC-Mass Spectrometer, model 5790A/5970A, used in the selected ion mode (SIM) with a DB-1MS 12-m × 0.2 mm × 0.33 μm cross-linked methyl silicone capillary column). The conditions were similar to those described previously [20]: splitless mode, 0.7 kg/cm^2 ^helium, 230°C injection temperature, column temperature at 150°C (initial) to 230°C at 20°/min, and scan speed of 690 amu/sec at an electron multiplier setting of 2,200 V. Underivatized authentic standards at various concentrations as well as samples were brought up in 10 μl MeOH or CH_2_Cl_2_, and 5 μl was injected for each GC/MS analysis. The authentic steroids were first run in standard mode, which showed that, under the condition employed, 5αP and progesterone each eluted as single peaks (at about 12.5 and 14.2 minutes, respectively), and 3αHP eluted as three separate peaks consisting of two isomeric dehydrated moieties and whole 3αHP (at 7.6, 8.3, and 11.1 minutes, respectively) (see Additional file 1A). The runs in standard mode also showed that the major ions (*m/e*) were 246, 283, 298, and 316, for 3αHP and its two dehydrated fragments; 231, 258, 298, and 316 for 5αP; and 124, 229, 272, and 314 for progesterone (Additional file 1B through E). Authentic steroids and sample extracts were then run in SIM set for the respective major ions. Quantification of samples was calculated by comparison with concentration curves developed from standards (range of 0.04 to 20 ng), and the limit of detection was about 50 pg.

### Statistical analyses

Statistical analyses were carried out with GraphPad Instat software (Graph-Pad Software, Inc., San Diego, CA, USA). Results are presented as mean ± SEM and were analyzed with the unpaired Student *t *test, with *P *< 0.05 considered statistically significant.

## Results

**Figure 2:**
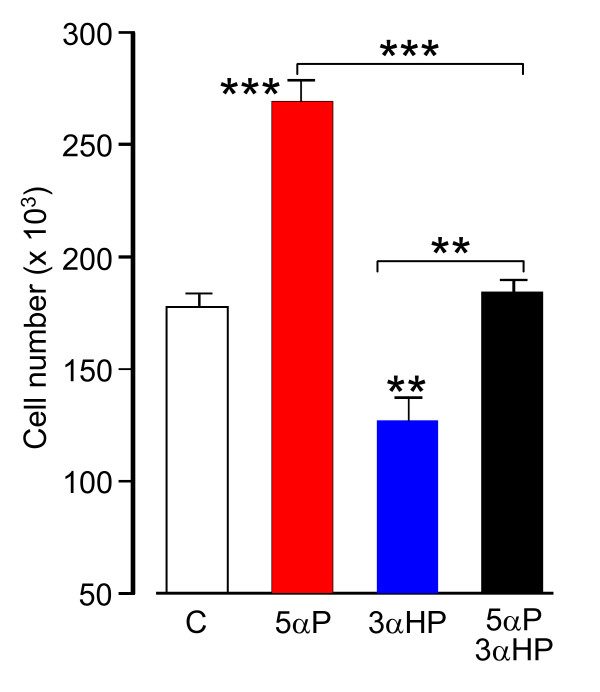
**Opposing *in vitro *effects of 5αP and 3αHP on proliferation of MDA-MB-231 cells used in the *in vivo *(xenograft) studies**. Cells were seeded at 4 × 10^4 ^cells per dish, allowed to attach for 24 hours, and then treated for 72 hours without (C, control) or with 10^-6 ^*M *5αP and/or 3αHP, and proliferation was determined by cell counts. Data are presented as cell number (mean and SEM; *n *= 4). **P < 0.01, ***P < 0.001 for the indicated comparisons or versus the control.

The human breast cell line MDA-MB-231 was chosen because these ER/PR-negative cells have been shown to exhibit opposing responses to 5αP and 3αHP *in vitro *[18], and because they have the capacity to form tumors spontaneously (without estrogen or progesterone supplements) when implanted into the mammary fat pad of immunodeficient mice [24]. The latter feature was considered important to study the potential of both stimulation and suppression of tumorigenesis. *In vitro *tests confirmed that proliferation of the passages of MDA-MB-231 cells used in the animal trials was significantly stimulated by 5αP and suppressed by 3αHP (Figure 2).

### ER/PR-negative breast cell tumorigenesis and tumor growth are stimulated by 5αP and suppressed by 3αHP

**Figure 3:**
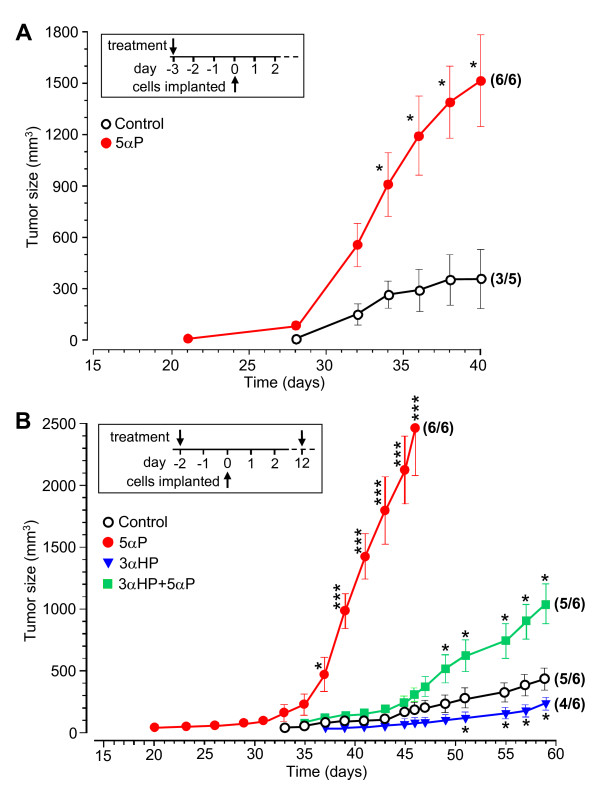
**ER/PR-negative breast cell tumor induction and growth are regulated by 5αP and 3αHP**. **(A) **Tumor induction and growth are stimulated by 5αP. MDA-MB-231 cells were implanted in mammary fat pads of 11 mice (day 0, inset); 3 days before (day -3), five mice were injected with vehicle (control; black open circles), and six were injected with 5αP (red, solid circles). Data points represent size (mm^3^; mean ± SEM) of tumors that developed of a total number of mice per treatment (bracketed values), and the experiment was terminated on day 40. *Significantly different from controls at *P *< 0.05. **(B) **Tumor induction and growth are stimulated by 5αP and inhibited by 3αHP. Twenty-four mice were divided into four groups of six mice each, and MDA-MB-231 cells were implanted on day 0 (inset). Two days before (day -2) and on day 12, mice were injected with either vehicle (control; black open circles), 5αP (red, solid circles), 3αHP (blue inverted triangles), or 5αP+3αHP (green squares). The 5αP-treated mice were terminated on day 46 because of tumor burden, and the other mice were terminated on day 59. Data points represent size (mm^3^; mean ± SEM) of tumors that developed of a total number of mice per treatment (bracketed values). Significantly different from controls at *P < 0.05 and ***P < 0.001.

To test the potential of 5αP to stimulate ER/PR-negative breast tumor formation and growth, 11 mice, 6 weeks old, were divided into two groups, five controls and six treated (Figure 3A). Three days (day -3) before cell implantation, they received a single injection of either vehicle (controls) or vehicle containing 5αP (Figure 3A, inset). The day of cell inoculation was considered as day 0, and the trial was terminated on day 40. In three of five controls and in six of six 5αP-treated mice, tumors developed. In the controls, small palpable tumors were first detected in two mice on day 28 and in the third mouse on day 32. Of the 5αP-treated mice, two had palpable tumors on day 21, and all six, by day 27. The tumors in the six 5αP-treated mice grew more rapidly and were on average 4.2-fold larger at termination than the three tumors in the control mice (*P *< 0.05). The results showed that incidence, onset, and growth of ER/PR-negative human breast cell tumors are stimulated by the progesterone metabolite, 5αP.

To determine whether 5αP and 3αHP have opposing actions on ER/PR-negative breast cell tumor formation, 24 mice were divided into four groups of six mice each and injected twice (on day -2 and day 12) with vehicle (control), or vehicle containing either 5αP, 3αHP, or 5αP+3αHP (Figure 3B). In the control group, a palpable tumor was observed in one mouse on day 33 and in four others by day 39, and the tumor size (mean ± SEM) was 164.4 ± 40.2 mm^3 ^on day 46 and 409.8 ± 73.8 mm^3 ^at termination (day 59). In the 5αP-treated group, two mice had palpable tumors by day 20, four by day 23, and all six by day 33; the tumors developed very rapidly, so that by day 46, the tumor volume was 2,431.8 ± 374.4 mm^3 ^(14.8-fold larger than in the controls; *P *< 0.001), and the tumor burden required termination of this group. In the 3αHP-treated group, palpable tumors were detected in two mice on day 37 and in two other mice by day 41, with average size of 69.2 ± 6.6 mm^3 ^on day 46, and 159.6 ± 32.1 mm^3 ^on day 59, significantly smaller (*P *< 0.05) than those in the controls. In the group treated with 3αHP+5αP, palpable tumors were detected in two mice on day 35 and in three more on day 39, with average size of 351.6 ± 43.3 mm^3 ^on day 46, and 1,020 ± 140 mm^3 ^on day 59. In comparison with the 5αP-only treated group, the results of the combined treatment (3αHP+5αP) showed that 3αHP significantly suppressed the 5αP-induced onset and growth of tumors (*P *< 0.001); tumor volume was 11.3-fold smaller than in the 5αP-only group on day 46. Conversely, 5αP significantly countered the suppressive action of 3αHP in the 5αP+3αHP group (*P *< 0.01); the tumor volume in the combined-treatment group was 4.7-fold larger than that in the 3αHP-only treated group on day 59. The experiment was repeated (see Additional file 3), and the overall results confirmed that 5αP treatment stimulated, whereas 3αHP inhibited, tumor initiation and growth, whereas simultaneous treatment with both hormones (3αHP+5αP) resulted in abrogation of the effects of either hormone alone.

### 3αHP results in suppression and regression of 5αP-stimulated ER/PR-negative breast tumors

**Figure 4:**
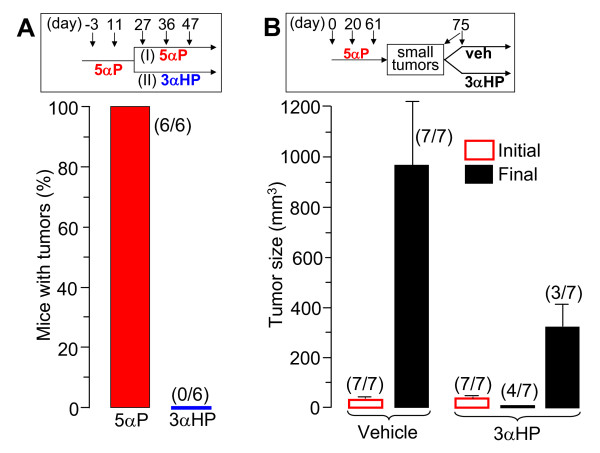
**3αHP results in suppression and regression of 5αP-induced ER/PR-negative tumors**. **(A) **3αHP suppresses ER/PR-negative breast cell tumorigenesis in 5αP-pretreated mice. Fourteen mice were treated with 5αP on day -3 and day 11 and then were divided into two groups. One group (Group I) continued to be treated with 5αP, whereas the other group (Group II) was treated with 3αHP on days 27, 36, and 47 (Inset). One mouse from each group was excluded from the final analysis, as explained under Results. The data are presented as the percentage of mice with tumors at termination. **(B) **3αHP results in regression of 5αP-induced ER/PR-negative breast cell tumors. Twenty-four mice with MDA-MB-231 cell implants received injections of 5αP on days 0, 20, and 61 (inset); on day 75, the 14 mice with approximately similar-sized small palpable tumors (18 to 34 mm^3^) were divided into two groups, consisting of seven mice each, which received a single injection of either vehicle (veh) or 3αHP, and the experiment was terminated 24 days later. Bars represent size (mm^3^; mean ± SEM) of tumors that developed of a total number of mice per treatment (bracketed values), at the start of treatments (day 75, Initial) and at termination (day 99, Final).

These experiments showed that the stimulatory effects of 5αP and the inhibitory effects of 3αHP are abrogated when the two hormones are given simultaneously, starting near the time of cell implantation. Two experiments were conducted to determine whether 3αHP can suppress and/or reverse the tumorigenic effects initiated by prior treatments with 5αP. The first experiment (Figure 4A) was conducted to determine if multiple treatments with 3αHP over an extended period result in a higher level/incidence of suppression of tumorigenesis. Fourteen mice were treated with 5αP on day -3 and day 11, and then seven of these mice continued to be treated with 5αP (Group I), whereas the other seven (Group II) were treated with 3αHP on days 27, 36, and 47 (Figure 4A, inset). Two mice were excluded from the final analysis: one from Group I, which failed to develop a tumor, and one from Group II, which started to develop an aggressive tumor just 9 days after the first treatment with 3αHP (about 5 to 6 weeks before the onset of any other tumor). At termination (day 96), all (six of six) of Group I mice had tumors (1,034.4 ± 399.1 mm^3^), and none (none of six) of Group II mice had tumors, indicating more-marked suppression of 5αP-stimulated tumorigenesis by multiple 3αHP treatments.

The second experiment (Figure 4B) was conducted to determine whether 3αHP can reverse the tumorigenic effects initiated by prior treatments with 5αP. Twenty-four mice received three subcutaneous injections (days 0, 20, and 61) of 5αP. Then, on day 75, 14 mice with similar-sized small palpable tumors (18 to 34 mm^3^) were divided into two groups, consisting of seven mice each, which received one injection of either vehicle (control) or 3αHP (Figure 4B, inset). At termination 24 days later, all seven of the control mice had enlarged tumors (950.9 ± 277.6 mm^3^), whereas of the seven 3αHP-treated mice, four had regressed to either no palpable tumors or just tiny nodules (≤5 mm^3^), and three had relatively small tumors (333.7 ± 90.1 mm^3^), indicating marked suppression and regression of 5αP-induced tumors by treatment with 3αHP.

### Effect of treatments on health and condition of animals and on tumor histopathology

**Figure 5:**
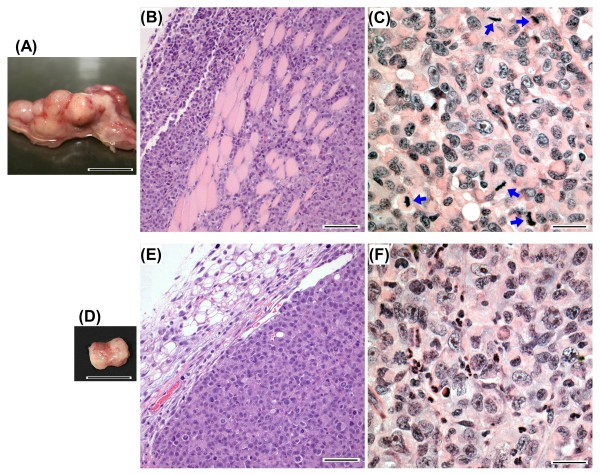
**Examples of MDA-MB-231 tumors and tumor histology from 5αP- and 3αHP-treated mice**. **(A) **Large aggressive tumor from a 5αP-treated mouse, with histologic sections showing **(B) **invasion of rib-cage muscle by the spreading tumor cells, and **(C) **higher magnification of region with numerous mitoses (arrows). **(D) **Residual tumor from a 3αHP-treated mouse, with no signs of invasion **(E) **and showing region of tumor with numerous apoptotic and necrotic cells **(F)**. Formalin-fixed sections (5 μm) stained with hematoxylin and eosin. Scale bars at 1.0 cm for whole tumors (A, D), at 160 µm for (B) and (E), and at 40 µm for (C) and (F).

At termination of experiments, mice from all treatment groups appeared to be in good body condition; no significant differences were noted in body-weight gain and in general appearance and condition of liver, lung, kidney, heart, and adrenals between control mice and mice from different treatments. Mice with enlarged tumors were observed to have some enlargement of the pancreas and spleen. The large 5αP-stimulated tumors tended to extend from the right ventral mammary fat pad area and laterally to the dorsal thoracic area near the steroid depots (Figure 5A). Histopathologic analyses of tumors from 5αP-treated mice showed hypercellular solid carcinomas invading the muscle and other outer aspects of the thoracic cavity (Figure 5B) and exhibited frequent mitoses (Figure 5C). In contrast, residual tumors in 3αHP-treated mice (Figure 5D) showed little or no invasion into surrounding tissue (Figure 5E) and generally exhibited less-frequent mitoses and more-frequent multifocal necroses (Figure 5F).

### Concentrations of 5αP and 3αHP in serum and tumors

#### Validation of RIA measurements by mass spectrometry

To determine the levels of 5αP, 3αHP, and progesterone in serum and tumors, RIAs specific for these steroids were used. For validation of the RIA measurements, aliquots of TLC-separated 5αP, 3αHP, and progesterone extracts from four tumors were tested with both RIA and gas chromatography-mass spectrometry (GC-MS). The results showed no significant differences between RIA and GC-MS measurements of hormone levels (see Additional file 2) and provided validation of the reliability of the RIA measurements.

#### Serum levels of 3αHP and 5αP are elevated after hormone treatments

**Figure 6:**
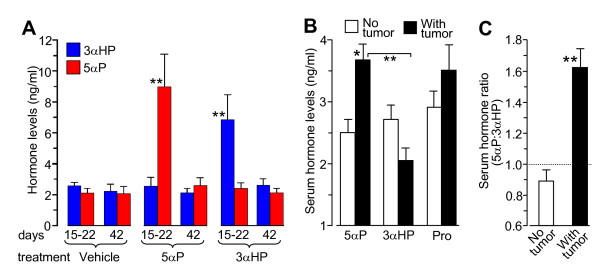
**Hormone levels in serum**. **(A) **Serum hormone levels after treatment. Serum samples (*n *= 4 to 5) were analyzed 15 to 22 days and 42 days after mice received an injection of vehicle (control), 5αP, or 3αHP. Data points represent nanograms per milliliter (mean ± SEM). (**P < 0.01 compared with respective controls). **(B) **Hormone levels in serum from 10 control (vehicle only) mice, six of which had developed tumors spontaneously (With tumor), and four that remained without tumors (No tumor). Hormone levels were determined with RIA and are presented as nanograms per milliliter 5αP, 3αHP, and progesterone (Pro) and **(C) **as the ratio of 5αP to 3αHP. *P < 0.05; **P < 0.01 for the comparison, as indicated or versus the levels in the "No tumor" mice.

To determine whether steroid treatments resulted in increased concentrations in the circulation, serum samples from vehicle (control) and hormone-injected mice were analyzed for 5αP and 3αHP at 15 to 22 days and 42 days after the last treatment (Figure 6A). Although 5αP and 3αHP concentrations were about the same (2 to 3 ng/ml) at both time periods in the vehicle-injected mice, the concentrations were significantly higher (about threefold to sixfold) in treated mice at 15 to 22 days after hormone injections and had declined to control levels by 42 days.

#### Levels of 5αP are higher in serum from control mice with tumors than in those without tumors

Measurements of hormone levels in serum of control animals (vehicle only) at termination showed that the 5αP concentrations were significantly higher (*P *< 0.05) in mice with tumors than in mice without tumors (Figure 6B). Also, in serum from control mice with tumors, the concentrations of 5αP were significantly higher (*P *< 0.01) than the concentrations of 3αHP, resulting in significantly higher 5αP/3αHP ratios (*P *< 0.01) than in serum from mice without tumors (Figure 6C).

#### Tumors have higher levels of 5αP than 3αHP and higher 5αP/3αHP concentration ratios than serum

**Figure 7:**
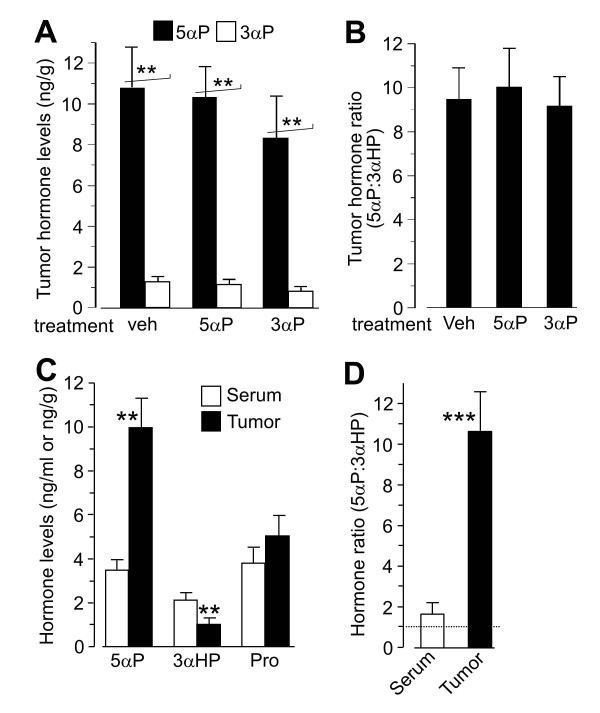
**Hormone levels in tumors**. **(A) **Effect of hormone treatment on 5αP and 3αHP levels in tumors. Hormone levels in tumors from four vehicle-injected, five 5αP-treated, and four 3αHP-treated mice were determined with RIA, as described in Methods. Hormone levels are presented as nanograms per milliliter 5αP and 3αHP and **(B) **as the ratio of 5αP to 3αHP. (**P < 0.01 for the comparison between 5αP and 3αHP levels). **(C) **Levels of 5αP, 3αHP, and progesterone (Pro) in tumors and respective sera from nine mice were determined after extraction with organic solvents and separation by thin-layer chromatography (TLC) as described in Methods. Levels of hormones are presented as nanograms per milliliter (serum) and nanograms per milligram (tumors), and **(D)**, as the ratio of 5αP to 3αHP. **, ***Significantly different from serum levels at *P *< 0.01 and *P *< 0.001, respectively.

To determine whether levels of 5αP and 3αHP in tumors are affected by hormone treatments, tumors from mice that had received vehicle, 5αP, or 3αHP injections before tumor initiation were analyzed at termination (Figure 7A). The results showed no significant differences in tumor hormone levels due to treatments. All the tumors had significantly higher levels of 5αP (about 10-fold) than 3αHP (*P *< 0.01), and the 5αP/3αHP ratios were not significantly different between tumors from mice with different treatments (Figure 7B). To compare hormone concentrations in tumors and respective sera, levels of 5αP, 3αHP, and progesterone were determined in samples from nine tumorous mice (Figure 7C). The levels of 5αP were significantly higher (*P *< 0.01), and those of 3αHP were significantly lower (*P *< 0.01) in tumors than in serum, whereas the progesterone levels did not differ significantly. In tumors, the concentrations of 5αP were on average more than 10-fold higher than those of 3αHP, and the 5αP/3αHP ratios were more than fivefold greater than in the respective sera (Figure 7D; *P *< 0.001). Significantly higher levels of 5αP than of 3αHP were also confirmed in samples from the four tumors analyzed with mass spectrometry (Additional file 2).

## Discussion

Estrogens and progesterone have long been considered to play cardinal roles in breast cancer, and therefore, expressions of ER and PR are widely used as indicators of hormonal dependency [4,25] and as determinants for current hormone-based treatments [26]. The relevance of the steroid receptors is highlighted by observations that ER/PR-negative breast cancer cells such as MDA-MB-231, which normally do not respond to estrogen and progesterone, can become responsive, both *in vitro *and *in vivo*, if they are transfected with the receptors [27,28]. Thus, the prevailing theory of hormonal regulation of breast cancer, as well as hormone-based therapies, revolves around estrogen and/or progesterone and ER/PR-positive breast cells and tumors. However, a large percentage of breast tumors are ER and/or PR negative [4,6-8] and therefore are not explained by the actions of estrogen and/or progesterone. Not only do these "receptor-negative" breast cancers fail to benefit from current hormonal therapies, but they also generally exhibit more-aggressive biologic behaviors and poorer prognosis than the receptor-positive ones [10-14]. The results of the studies reported here show for the first time that the progesterone metabolites, 5αP and 3αHP, act as hormones that regulate ER/PR-negative breast tumor formation, growth, and regression. The onset of the ER/PR-negative human breast cell tumors in mice was considerably accelerated, and the growth significantly stimulated, by just one or two applications of 5αP. In contrast, 3αHP retarded onset of tumor formation, suppressed tumor growth, and inhibited or regressed existing 5αP-induced tumors. When both hormones were administered simultaneously, the effects of one were abrogated by the effects of the other. The current *in vivo *demonstrations, from five separate experiments, of the opposing actions of 5αP and 3αHP on tumorigenesis and tumor growth extend the previous findings, which showed opposing *in vitro *cancer-regulatory actions of these progesterone metabolites on receptor-negative (MDA-MB-231, MCF-10A) as well as receptor-positive (MCF-7, T47D, ZR-75-1) breast cell lines [15,17,18].

The response of ER/PR-negative breast cells to the progesterone metabolites can be explained by the presence of specific high-affinity receptors for 5αP (5αPR) and 3αHP (3αHPR). The 5αPR and 3αHPR (which are associated with the plasma membranes of both ER/PR-positive [19] and ER/PR-negative [29] cells) are distinct from each other and from known ER, PR, androgen, and corticosteroid receptors, and lack affinity for other steroids, such as progesterone, estrogen, androgens, corticosteroids, and other progesterone metabolites [19]. Levels of 5αPR are upregulated by 5αP itself and estradiol, and downregulated by 3αHP in both ER/PR-positive and -negative cells [29]. The mechanisms of action resulting in the opposing effects of these two hormones appear to involve cell-signaling pathways associated with the plasma membrane receptors, as well as altered gene expression. Indications are that 5αP acts via the surface receptor-linked mitogen-activated protein kinase (MAPK; Erk1/2) pathway; 5αP significantly stimulates activation of Erk1/2 [30], increases the Bcl-2/Bax expression ratio [18] and actin depolymerization [31], and decreases expression of actin and adhesion plaque-associated vinculin [31], resulting in decreased apoptosis and increased mitosis and cell detachment. Conversely, 3αHP appears to suppress protein kinase C (PKC), phospholipase C (PLC), Ca^2+ ^mobilization (unpublished observations), and the Bcl-2/Bax expression ratio [18], and increases expression of the cell-cycle inhibitor p21 [18], resulting in increased apoptosis and decreased proliferation and detachment of breast cell lines. In pituitary cells, 3αHP also has been shown to inhibit a plasma membrane-associated PKC, PLC, Ca^2+ ^cell-signaling pathway [32].

The results of the studies reported here not only show that 5αP and 3αHP have opposing effects on initiation and growth of ER/PR-negative human breast tumors, but also provide *in vivo *evidence of the marked changes in the relative concentrations of these hormones in the tumor microenvironment. Whereas serum from control mice, in which implanted human breast cells had not developed into tumors, contained about equal concentrations of 5αP and 3αHP, serum from mice with tumors had significantly more 5αP than 3αHP. Because hormones had not been administered to these mice, the higher 5αP/3αHP ratio in serum from tumor-bearing mice can reasonably be expected to have resulted from the tumors, which on average had about threefold higher concentrations of 5αP than the respective sera, and >10-fold higher 5αP than 3αHP levels. Previous *in vitro *metabolism studies showed that human breast tumor tissues convert significantly more progesterone to 5α-pregnanes like 5αP and less to 4-pregnenes like 3αHP than do paired normal (nontumorous) tissues [15] and that these differences correlated with significantly higher 5α-reductase gene (*SRD5A1, SRD5A2*) and lower 3α(20α)-HSO gene (*AKR1C1, AKR1C2, AKR1C3*) expression in tumor tissues [33]. Similar differences in progesterone metabolism and enzyme gene expressions were observed between tumorigenic and nontumorigenic breast cell lines [16]. In addition to the ability to convert progesterone to active cancer-regulating hormones, breast carcinomas are able to synthesize progesterone [34,35], which could account for its relatively high concentrations in the xenograft tumors reported here, and indicate an *in situ *supply of the biosynthetic precursor of 5αP and 3αHP.

**Figure 8:**
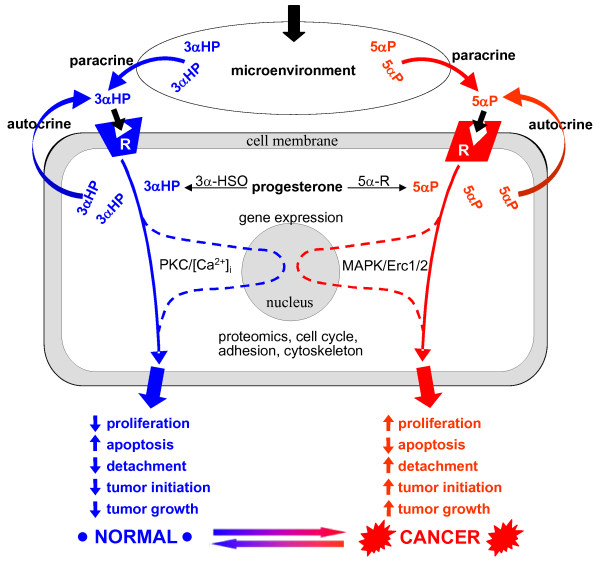
**Summary of the opposing autocrine/paracrine effects of the progesterone metabolites, 5αP and 3αHP, in a stylized ER/PR-negative human breast cell**. Evidence presented here shows that a high concentration of 5αP relative to 3αHP, in the microenvironment, promotes initiation and growth of ER/PR-negative human breast cell tumors, whereas a higher concentration of 3αHP, relative to 5αP, suppresses tumorigenesis and promotes normalcy. Progesterone is converted to 3αHP and 5αP in breast cells. Tumorigenic and tumor cells convert more progesterone to 5αP and less to 3αHP than do normal cells. The steroids, being lipophylic, are able to pass out of cells and result in a concentration buildup in the microenvironment. The result is a significant increase in the 5αP-to-3αHP concentration ratio in the microenvironment of tumorigenic cells and within tumorous tissues in comparison with normal (nontumorous) breasts. 3αHP and 5αP bind to specific receptors on the plasma membrane linked to signaling pathways involving PKC, phospholipase C, and Ca^2+ ^mobilization (3αHP) and MAPK/Erk1/2 (5αP) and to modulators of gene expression. The cancer-inhibiting actions of 3αHP result in decreased proliferation and detachment of cells, increased apoptosis, and suppression of tumor initiation and growth. The cancer-promoting actions of 5αP have the opposite effects and result in stimulation of tumorigenesis and tumor growth. The evidence suggests that high concentrations of 5αP relative to 3αHP in the microenvironment will promote progression toward neoplasia and tumorigenesis, whereas a low 5αP-to-3αHP concentration ratio favors maintenance of the normal state.

The significant concentrations of 5αP and 3αHP, and particularly the high 5αP/3αHP ratios, in the MDA-MB-231 xenograft tumors, emphasize the potential importance of the microenvironment within breast tissue where the biologic actions occur. The role of the microenvironment in changing the expression of regulatory factors such as metabolizing enzymes, receptors, cytoskeletal and adhesion molecules, and growth promoters/inhibitors and in epigenetic alterations has been extensively reviewed [36-40]. The current findings, along with the previous *in vitro *studies, suggest that the relative concentrations of 5αP and 3αHP in the breast microenvironment constitute important autocrine/paracrine determinants not only for tumorigenesis but also for potential regression of tumors and the maintenance of normalcy of ER/PR-negative breast cells/tissues. Figure 8 provides a summary of opposing biologic actions and proposed mechanisms of action of the progesterone metabolites, 5αP and 3αHP, in promoting neoplasia and tumorigenesis, as well as in maintaining normalcy in ER/PR-negative human breast cells. Evidence presented here shows that a high concentration of 5αP, relative to 3αHP in the microenvironment, promotes initiation and growth of tumors, whereas a higher concentration of 3αHP, relative to 5αP, suppresses tumorigenesis and promotes normalcy. Previous evidence indicates that these opposing effects of 5αP and 3αHP are propagated via the opposing actions of the hormones on cell proliferation (mitosis, apoptosis), adhesion, cell cycle, regulatory and signaling molecules, and gene expression after binding to specific receptors.

How might higher levels of either 5αP or 3αHP in the serum due to the steroid implants have, respectively, initiated/promoted or suppressed xenograft tumorigenesis? The 5αP and 3αHP treatments, consisting of suspensions placed subcutaneously in the nape of the neck, resulted in elevated serum levels of either hormone, which persisted for about 2 to 3 weeks after the last injection. Because the depots were not far removed from the site of the cell implants, lymph drainage may have resulted in significantly higher concentrations of each applied hormone in the immediate vicinity of the implanted human cells. Conceivably, in the 5αP-treated mice, the induced elevation of 5αP levels, relative to 3αHP, in the microenvironment of the human cell implants, could have exerted procancer actions that initiated tumorigenesis. Because 5α-reductase and 5αPR levels are upregulated by 5αP [29], the *in situ *production and paracrine/autocrine actions of locally elevated 5αP could then have autoenhanced hormone-receptor interaction and the resulting stimulation of tumor growth, as illustrated in Figure 8. In a like manner, in the 3αHP-treated mice, the elevated 3αHP levels, relative to 5αP, in the microenvironment could have opposed progression to xenograft neoplasia by its inherent anticancer actions and the suppression of 5αP synthesis and 5αPR expression [29]. By extension, in an intact human breast, local changes in relative concentrations of 5αP and 3αHP (that is, changes in the 5αP/3αHP ratio) resulting from selective up- or downregulation of progesterone-metabolizing enzymes induced by microenvironmental triggers could determine ER/PR-negative breast cell progression to tumor initiation and growth or maintenance of normalcy. Because only small changes in enzyme activity/expression are needed to result in significant local concentration changes, either a slight elevation of 5α-reductase, or a reduction of 3α-HSO, in one or more cells could lead to an increase in the ratio of 5αP/3αHP in the immediate intra- and extracellular environment. Conversely, processes that result in higher levels of cancer-suppressing 3αHP (and consequently lower 5αP/3αHP ratios) could ensure maintenance of normalcy.

Because *in vitro *studies have shown that both ER- and/or PR-negative and -positive breast cells respond in a stimulatory and inhibitory fashion, respectively, to 5αP and 3αHP, and have 5αPR and 3αHPR, the present results also may have implications for the substantial numbers (20% to 40%) of ER/PR-positive patients who fail to respond to suppression of estrogen and/or progesterone levels or actions [4,41,42], as well as for those receptor-positive tumors that do respond to hormonal therapies. In this regard, the opposing actions of the progesterone metabolites also appear to exert some control over the estrogen-regulated effects on breast cancer by their ability to modulate ER numbers in ER-positive cells [43]. Furthermore, because both tumorigenic and normal (nontumorigenic) breast cells respond to the opposing actions of 5αP and 3αHP, the relative concentrations of the progesterone metabolites in the microenvironment may also play a role in maintaining normalcy of breast tissues in general, regardless of ER/PR status.

## Conclusions

*In vivo *evidence shows that the progesterone metabolites, 5αP and 3αHP, control tumorigenesis of ER/PR-negative human breast cells: 5αP stimulates, whereas 3αHP suppresses, initiation and growth of tumors. Although both hormones can be synthesized by breast cells/tissues, tumors produce significantly more 5αP than 3αHP, resulting in high 5αP/3αHP ratios in the breast microenvironment. The findings provide the first hormonal explanation of the regulation of ER/PR-negative breast tumors. The results suggest new hormonal biomarkers, diagnostics, and therapeutics for these aggressive "receptor-negative" breast tumors that are unresponsive to current hormonal therapies. Moreover, because both ER/PR-negative and ER/PR-positive, as well as normal and tumorigenic human breast cell lines, have been shown to respond to 5αP and 3αHP *in vitro*, it is suggested that these endogenously produced progesterone metabolites may also play regulatory hormonal roles in ER/PR-positive breast cancers, as well as in the maintenance of normalcy in nontumorous breast tissues. The *in vivo *data provide further evidence that progesterone metabolites, such as 5αP and 3αHP, deserve to be considered as active hormones in their own right, rather than inactive waste products, and that they must be considered in the development of new approaches to prevention, detection, and treatment of breast cancers.

## Abbreviations

5αP: 5α-pregnane-3:20-dione (5α-dihydroprogesterone); 3αHP: 4-pregnen-3α-ol-20-one (3α-dihydroprogesterone); 5αPR: 5αP receptor; 3αHPR: 3αHP receptor; ER: estrogen receptor; GC-MS: gas chromatography mass spectrometry; HSO: hydroxysteroid oxidoreductase; HPLC: high-performance liquid chromatography; MAPK: mitogen-activated protein kinase; MAPK/Erc: MAPK/extracellular signal-regulated kinase; PKC: protein kinase C; PLC: phospholipase C; PR: progesterone receptor; RIA: radioimmunoassay; TLC: thin-layer chromatography.

## Competing interests

The authors declare that they have no competing interests.

## Authors' contributions

JPW conceived of the study, created the study design, performed mass spectrometry and radioisotope ([^3^H5αP and [^3^H]3αHP) syntheses, prepared figures, and assisted in animal studies, data analyses, and hormone measurements, and drafted the manuscript. GZ helped in the study design, performed the *in vitro *proliferation studies, participated in the xenograft studies, hormone preparation, RIA measurements, and literature review. IW was involved in the xenograft studies, performed histopathologic analyses, prepared figures, and assisted in editing the manuscript. HTC carried out the animal studies, surgically implanted the cells, performed the hormone treatments, tumor necropsies, growth measurements and analyses, and helped in generating antibodies for 5αP used in the RIA. All authors read and approved the final manuscript.

## Supplementary Material

Additional file 1 **Mass spectrometry (GC-MS) of 3αHP, 5αP, and progesterone**. Click here for file

Additional file 2 **Comparison of hormone measurements with radioimmunoassay (RIA) and GC-MS**. Click here for file

Additional File 3 **Additional experiment showing the opposing effects of 5αP and 3αHP on ER/PR-negative breast cell tumorigenesis and growth**. (Similar to Figure 3). Click here for file

## References

[B1] American Cancer Society Global Cancer Facts & Figures 2011 2 Atlanta, American Cancer Society http://www.cancer.org/acs/groups/content/@epidemiologysurveilance/document/acsp-027766.pdf

[B2] Key TJ Pike MC The role of estrogens and progestogens in the epidemiology and prevention of breast cancer Eur J Cancer Clin Oncol 1988 24 29 43 10.1016/0277-5379(88)90173-3 3276531

[B3] Henderson BE Feigelson HS Hormonal carcinogenesis Carcinogenesis 2000 21 427 433 10.1093/carcin/21.3.427 10688862

[B4] McGuire WL Osborne CK Clark GM Knight WA III Steroid hormone receptors and carcinoma of the breast Am J Physiol 1982 243 E99 E102 7114210 10.1152/ajpendo.1982.243.2.E99

[B5] Aaltomaa S Lipponen P Eskelinen M Kosma VM Marin S Alhava E Syrjanen K Hormone receptors as prognostic factors in female breast cancer Ann Med 1991 6 643 648 1777219 10.3109/07853899109148097

[B6] Howat JMT Harris M Swindell R Barnes DM The effect of oestrogen and progesterone receptors on recurrence and survival in patients with carcinoma of the breast Br J Cancer 1985 51 263 270 10.1038/bjc.1985.38 3966982 PMC1977040

[B7] Taucher S Rudas M Gnant M Thomanek K Dubsky P Roka S Bachleitner T Kandioler D Wenzel C Steger G Mittlböck M Jakesz R Sequential steroid hormone receptor measurements in primary breast cancer with and without intervening primary chemotherapy Endocr Relat Cancer 2003 10 91 98 10.1677/erc.0.0100091 12653672

[B8] Rexhepaj E Brennan DJ Holloway P Kay EW McCann AH Landberg G Duffy MJ Jirstrom K Gallagher WM Novel image analysis approach for quantifying expression of nuclear proteins assessed by immunohistochemistry: application to measurement of oestrogen and progesterone receptor levels in breast cancer Breast Cancer Res 2008 10 R89 10.1186/bcr2187 18947395 PMC2614526

[B9] Robinson GW Hennighausen L Johnson PF Side-branching in the mammary gland: the progesterone-Wnt connection Genes Dev 2000 14 889 894 10783160

[B10] Paone JF Abeloff MD Ettinger DS Arnold EA Baker PR The correlation of estrogen and progesterone receptor levels with response to chemotherapy for advanced carcinoma of the breast Surg Gynecol Obstet 1981 152 70 74 7455895

[B11] Vollenweider-Zerargui L Barrelet L Wong Y Lemarchand-Béraud T Gómez F The predictive value of estrogen and progesterone receptors' concentrations on the clinical behavior of breast cancer in women: clinical correlation on 547 patients Cancer 1986 57 1171 1180 10.1002/1097-0142(19860315)57:6<1171::AID-CNCR2820570618>3.0.CO;2-X 3943040

[B12] Fisher B Redmond C Fisher ER Caplan R Relative worth of estrogen or progesterone receptor and pathologic characteristics of differentiation as indicators of prognosis in node negative breast cancer patients: findings from National Surgical Adjuvant Breast and Bowel Project Protocol B-06 J Clin Oncol 1988 7 1076 1087 2856862 10.1200/JCO.1988.6.7.1076

[B13] Bardou V-J Arpino G Elledge RM Osborne CK Clark GM Progesterone receptor status significantly improves outcome prediction over estrogen receptor status alone for adjuvant endocrine therapy in two large breast cancer data bases J Clin Oncol 2003 21 1973 1979 10.1200/JCO.2003.09.099 12743151

[B14] Dunnwald LK Rossing MA Li CI Hormone receptor status, tumor characteristics, and prognosis: a prospective cohort of breast cancer patients Breast Cancer Res 2007 9 R6 doi:10.1186/bcr 1639 10.1186/bcr1639 17239243 PMC1851385

[B15] Wiebe JP Muzia D Hu J Szwajcer D Hill SA Seachrist JL The 4-pregnene and 5α-pregnane progesterone metabolites formed in nontumorous and tumorous breast tissue have opposite effects on breast cell proliferation and adhesion Cancer Res 2000 60 936 943 10706108

[B16] Wiebe JP Lewis MJ Activity and expression of progesterone metabolizing 5α-reductase, 20α-hydroxysteroid oxidoreductase and 3α(β)-hydroxysteroid oxidoreductases in tumorigenic (MCF-7, MDA-MB-231, T-47D) and nontumorigenic (MCF-10A) human breast cancer cells BMC Cancer 2003 3 9 10.1186/1471-2407-3-9 12659654 PMC154104

[B17] Wiebe JP Progesterone metabolites in breast cancer Endocr Relat Cancer 2006 13 717 738 10.1677/erc.1.01010 16954427

[B18] Wiebe JP Beausoleil M Zhang G Cialacu V Opposing actions of the progesterone metabolites, 5α-dihydroprogesterone (5αP) and 3α-dihydroprogesterone (3αHP) on mitosis, apoptosis, and expression of BCL-2, Bax and p21 in human breast cell lines J Steroid Biochem Mol Biol 2010 118 125 132 10.1016/j.jsbmb.2009.11.005 19931389

[B19] Weiler PJ Wiebe JP Plasma membrane receptors for the cancer-regulating progesterone metabolites, 5α-pregnane-3,20-dione and 3α-hydroxy-4-pregnen-20-one in MCF-7 breast cancer cells Biochem Biophys Res Commun 2000 272 731 737 10.1006/bbrc.2000.2847 10860824

[B20] Wiebe JP Barr KJ Buckingham KD A radioimmunoassay for the regulatory allylic steroid, 3α-hydroxy-4-pregnen-20-one (3αHP) J Steroid Biochem Mol Biol 1991 38 505 512 10.1016/0960-0760(91)90339-7 2031864

[B21] Erlanger BF Borek F Beiser SM Lieberman S Steroid-protein conjugates: preparation and characterization of conjugates of bovine serum albumin with testosterone and with cortisone J Biol Chem 1957 228 713 727 13475354

[B22] Naar J Branaa P Chinain M Pauillac S An improved method for the microscale preparation and characterization of hapten-protein conjugates: the use of cholesterol as a model for nonchromophore hydroxylated haptens Bioconjugate Chem 1999 10 1143 1149 10.1021/bc990042g 10563786

[B23] Wiebe JP Deline C Buckingham KD Dave V Stothers JB Synthesis of the allylic gonadal steroids, 3α-hydroxy-4-pregnen-20-one and 3α-hydroxy-4-androsten-17-one, and of 3α-hydroxy-5α-pregnan-20-one Steroids 1985 45 39 51 10.1016/0039-128X(85)90064-9 4089911

[B24] Price JE Polyzos A Zhang RD Daniels LM Tumorigenicity and metastasis of human breast carcinoma cell lines in nude mice Cancer Res 1990 50 717 721 2297709

[B25] Osborne CK Yochmowitz MG Knight WA McGuire WL The value of estrogen and progesterone receptors in the treatment of breast cancer Cancer 1980 46 2884 2888 10.1002/1097-0142(19801215)46:12+<2884::AID-CNCR2820461429>3.0.CO;2-U 7448733

[B26] Ciocca DR Elledge R Molecular markers for predicting response to tamoxifen in breast cancer patients Endocrine 2000 13 1 10 10.1385/ENDO:13:1:1 11051041

[B27] Jiang S-Y Jordan VC Growth regulation of estrogen receptor-negative breast cancer cells transfected with complementary DNAs for estrogen receptor J Natl Cancer Inst 1992 84 580 591 10.1093/jnci/84.8.580 1556769

[B28] Lin VCL Eng AS Hen NE Ng EHL Chowdhury SH Effect of progesterone on the invasive properties and tumor growth of progesterone receptor-transfected breast cancer cells MDA-MB-231 Clin Cancer Res 2001 7 2880 2886 11555606

[B29] Pawlak KJ Zhang G Wiebe JP Membrane 5α-pregnane-3,20-dione (5αP) receptors in MCF-7 and MCF-10A breast cancer cells are up-regulated by estradiol and 5αP and down-regulated by the progesterone metabolites, 3α-dihydroprogesterone and 20α-dihydroprogesterone, with associated changes in cell proliferation and detachment J Steroid Biochem Mol Biol 2005 97 278 288 10.1016/j.jsbmb.2005.05.014 16154741

[B30] Wiebe JP Lewis MJ Cialacu V Pawlak KJ Zhang G The role of progesterone metabolites in breast cancer: potential for new diagnostics and therapeutics J Steroid Biochem Mol Biol 2005 93 201 208 10.1016/j.jsbmb.2004.12.003 15860263

[B31] Wiebe JP Muzia D The endogenous progesterone metabolite, 5α-pregnane-3,20-dione, decreases cell-substrate attachment, adhesion plaques, vinculin expression, and polymerized F-actin in MCF-7 breast cancer cells Endocrine 2001 16 7 14 10.1385/ENDO:16:1:07 11822829

[B32] Beck CA Wolfe M Murphy LD Wiebe JP Acute, nongenomic actions of the neuroactive gonadal steroid, 3α-hydroxy-4-pregnen-20-one (3αHP), on FSH release in perifused rat anterior pituitary cells Endocrine 1997 6 221 229 10.1007/BF02820496 9368676

[B33] Lewis MJ Wiebe JP Heathcote JG Expression of progesterone metabolizing enzyme genes (AKR1C1, AKR1C2, AKR1C3, SRD5A1, SRD5A2) is altered in human breast carcinoma BMC Cancer 2004 4 27 10.1186/1471-2407-4-27 15212687 PMC459223

[B34] Abul-Hajj YJ Iverson R Kiang DT Metabolism of pregnenolone by human breast cancer: evidence for 17alpha-hydroxylase and 17,20-lyase Steroids 1979 34 817 827 10.1016/0039-128X(79)90094-1 161434

[B35] Gunasegaram R Peh KL Loganath A Ratnam SS Expression of 3β-hydroxysteroid dehydrogenase-5,4-en isomerase activity by infiltrating ductal human breast carcinoma *in vitro* Breast Cancer Res Treat 1998 50 117 123 10.1023/A:1006009031839 9822216

[B36] Sutherland RM Cell and environment interactions in tumor microregions: the multicell spheroid model Science 1988 240 177 184 10.1126/science.2451290 2451290

[B37] Kenny PA Bissell MJ Tumor reversion: correction of malignant behavior by microenvironmental cues Int J Cancer 2003 107 688 695 10.1002/ijc.11491 14566816 PMC2933180

[B38] Hayashi S-I Yamaguchi Y Estrogen signaling pathway and hormone therapy Breast Cancer 2008 15 256 261 10.1007/s12282-008-0070-z 18818989

[B39] Hu M Polyak K Molecular characterization of the tumor microenvironment in breast cancer Eur J Cancer 2008 44 2760 2765 10.1016/j.ejca.2008.09.038 19026532 PMC2729518

[B40] Wilson C Holen I Coleman RE Seed, soil and secreted hormones: potential interactions by breast cancer cells with their endocrine/paracrine microenvironments and implications for treatment with bisphosphonates Cancer Treat Rev 2012 38 877 889 10.1016/j.ctrv.2012.02.007 22398187

[B41] Fuqua SA Where is the lesion in hormone-independent breast cancer? J Natl Cancer Inst 1992 84 Bethesda 554 555 10.1093/jnci/84.8.554 1556761

[B42] Murphy LC Mechanisms of hormone independence in human breast cancer In Vivo 1998 12 95 106 9575432

[B43] Pawlak KJ Wiebe JP Regulation of estrogen receptor (ER) levels in MCF-7 cells by progesterone metabolites J Steroid Biochem Mol Biol 2007 107 172 179 10.1016/j.jsbmb.2007.05.030 17683929

