# Supplementary material for: Progesterone metabolites regulate induction, growth, and suppression of estrogen- and progesterone receptor-negative human breast cell tumors
Source: Breast Cancer Res. 2013 May 11;15(3):R38. doi: 10.1186/bcr3422 (PMC3706910; doi:10.1186/bcr3422)
Supplement: Additional file 1 — Mass spectrometry (GC-MS) of 3αHP, 5αP, and progesterone. [file bcr3422-S1.PDF]

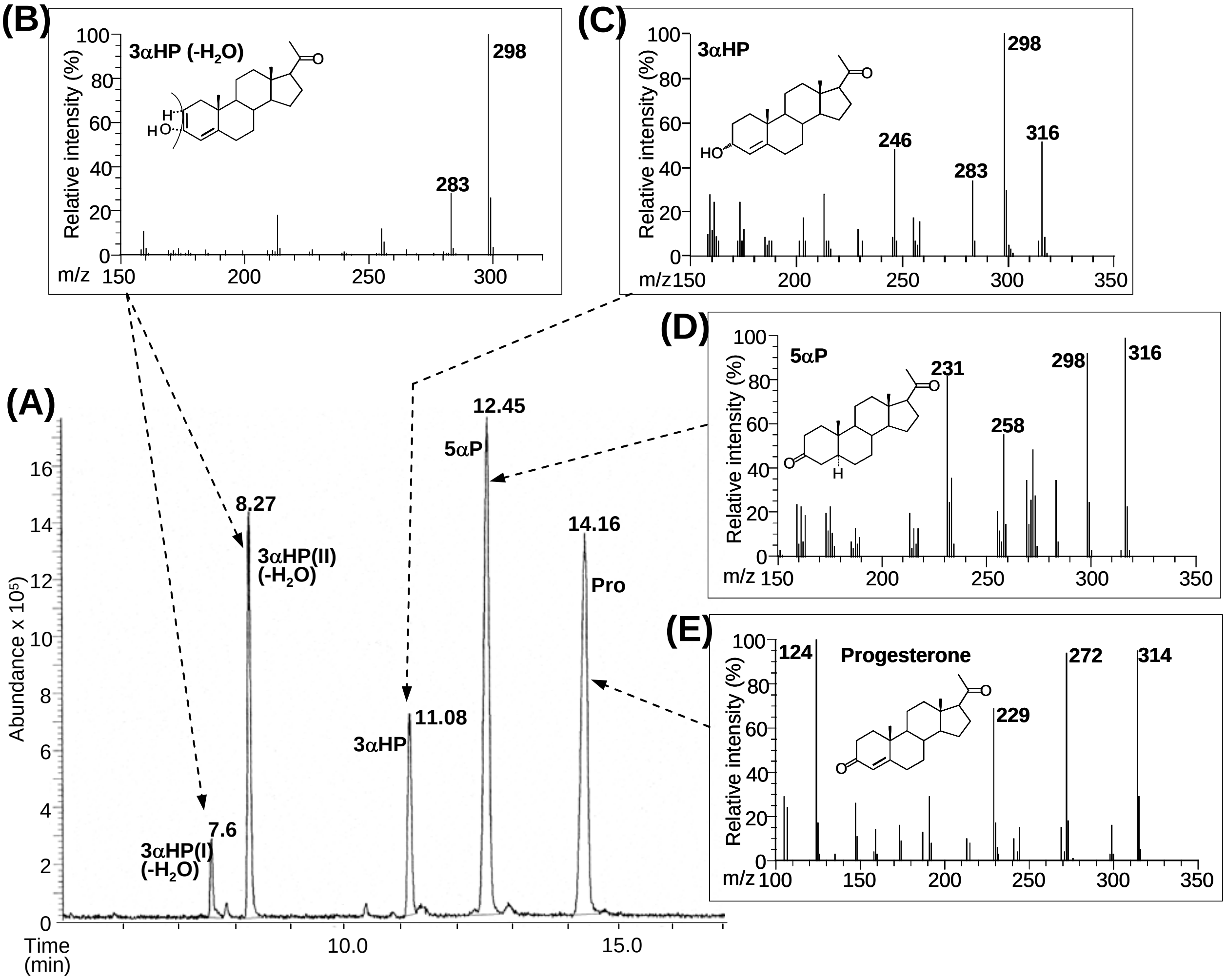

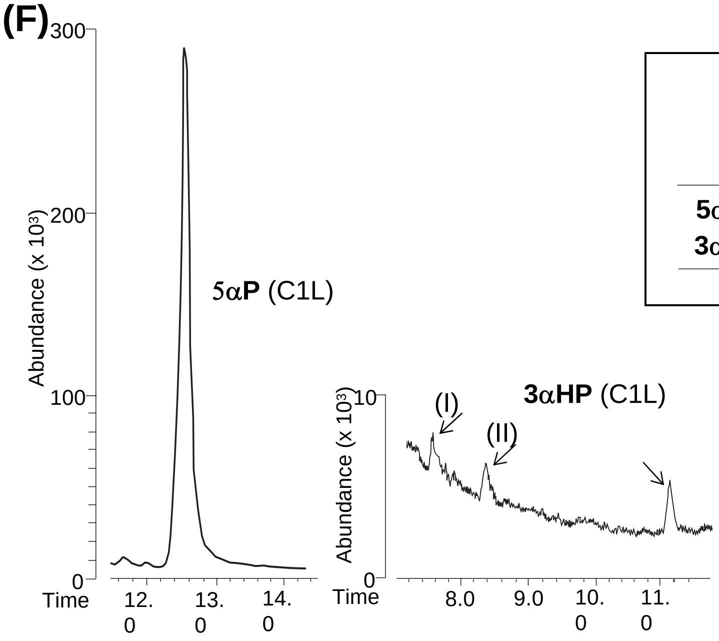

	Abundance (area units) (x10^6)	Abundance (ng)
5αP	18.954	3.63
3αHP	0.578	0.11

Additional file 1:

Gas chromatography mass spectrometry (GC-MS) of 3αHP, 5αP and progesterone standards and tumor extracts. Portions of purified extracts from four tumors were assayed by GC-MS in order to compare with the RIA results. **(A)** Separation of the standards at the GC-MS conditions employed (see Methods). 3αHP routinely appears as 3 peaks, as shown at retention times of 7.6, 8.27 and 11.08 min; peaks at 7.6 and 8.27 min are dehydrated (minus H_2O) isomeric 3αHP fragments with molecular mass of 298 and general ion spectrum shown in **(B)** and the peak at 11.08 min is the full 3αHP molecule, with molecular mass of 316 and the ion spectrum shown in **(C)**. The peaks at 12.45 min and 14.16 min are 5αP and progesterone, with ion spectra as shown in **(D)** and **(E)**, respectively. For quantification of samples, the GC-MS was run in selected ion mode (SIM), using the main ions (m/z) indicated in the spectra (b-e). Examples of traces from equal volumes of 5αP and 3αHP extracts from one tumor (CIL) are shown in **(F)**. Note that the ordinate scale for 5αP is 10 times larger than for 3αHP. In this example, the total integrated areas under the curve for the 5αP peak (18.954 x 10^6 units) and the combined 3αHP peaks (0.578 x 10^6 units) were calculated to be equivalent to 3.63 ng and 0.12 ng, respectively (Table, inset). GC-MS analyses of fractions from the other three tumors gave essentially similar results. RIA determinations were performed on the remaining fractions from each tumor and compared with the GC-MS determinations (see Additional file 2).
