# Supplementary material for: Progesterone metabolites regulate induction, growth, and suppression of estrogen- and progesterone receptor-negative human breast cell tumors
Source: Breast Cancer Res. 2013 May 11;15(3):R38. doi: 10.1186/bcr3422 (PMC3706910; doi:10.1186/bcr3422)
Supplement: Additional file 2 — Comparison of hormone measurements with radioimmunoassay (RIA) and GC-MS. [file bcr3422-S2.PDF]

Additional file 2.

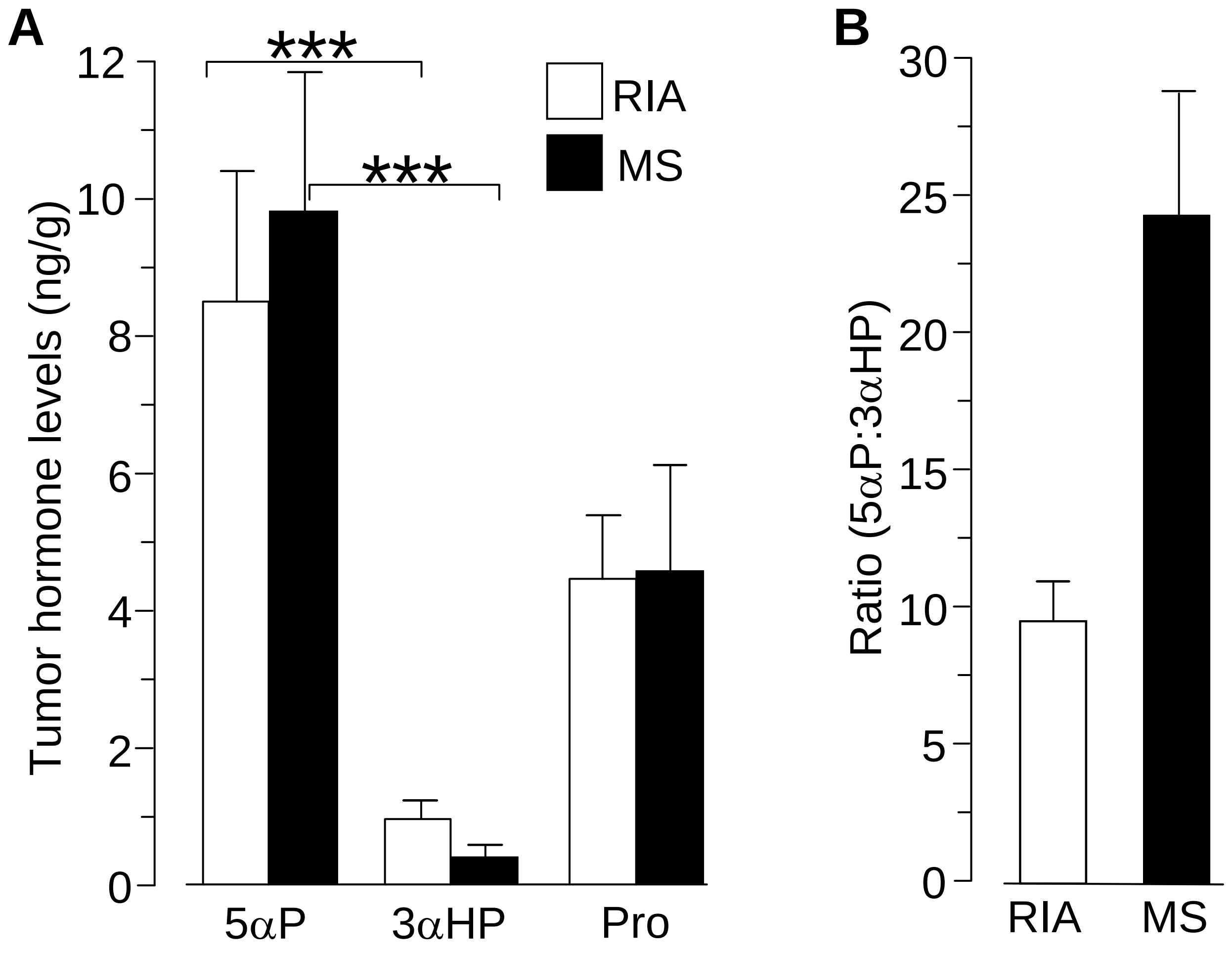

Additional file 2.

Comparison of tumor hormone measurements by RIA and gas chromatography mass spectrometry (GC-MS). To verify the RIA measurements, authentic standards as well as aliquots of TLC separated 5αP, 3αHP and progesterone (Pro) extracts from four tumors were tested by both RIA and GC-MS. The GC-MS was run on selected ion mode (SIM) set for the major ions as determined at conditions which provided good separation, spectral identity profiles and quantification of the three authentic steroids (see Methods and Additional file 1 for details). Levels are presented (mean and SEM) as ng/g **(A)** and as 5αP:3αHP ratio **(B)**. There were no significant differences between RIA and MS measurements. Both methods of measurement showed significantly higher levels of 5αP than 3αHP, resulting in high 5αP;3αHP ratios.
***P < 0.001 for the indicated comparisons.
