# Supplementary material for: Progesterone metabolites regulate induction, growth, and suppression of estrogen- and progesterone receptor-negative human breast cell tumors
Source: Breast Cancer Res. 2013 May 11;15(3):R38. doi: 10.1186/bcr3422 (PMC3706910; doi:10.1186/bcr3422)
Supplement: Additional File 3 — Additional experiment showing the opposing effects of 5αP and 3αHP on ER/PR-negative breast cell tumorigenesis and growth. (Similar to Figure 3). [file bcr3422-S3.PDF]

Additional file 3.

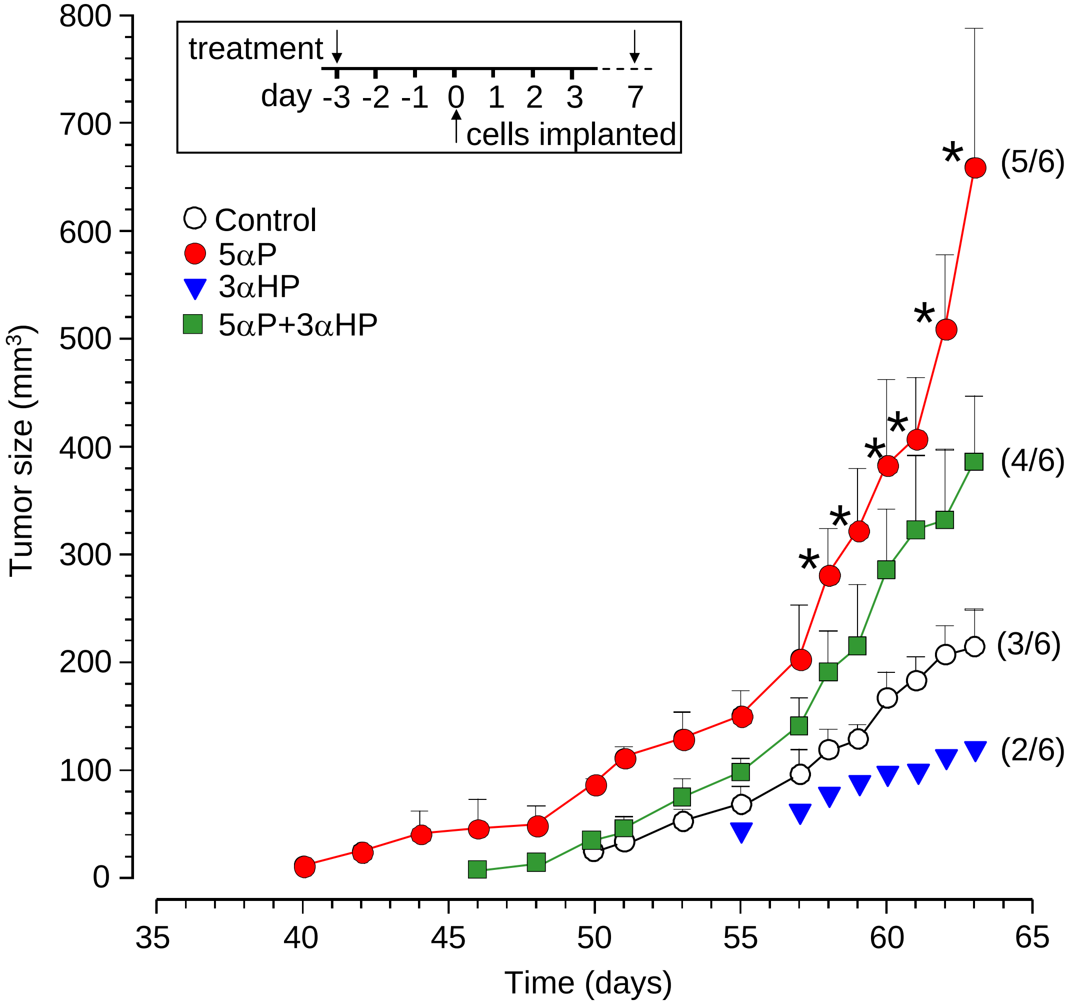

<u>Additional file 3</u>.
The opposing effects of 5αP and 3αHP on initiation and growth of ER/PR-negative breast cell tumors in a xenograft experiment essentially similar to that shown in text Figure 3b. Twenty-four mice were divided into four groups of six mice each and human breast cells were implanted on day 0; two days before (day -2) and on day 7, mice were injected with either vehicle (control), or 5αP, 3αHP, or 5αP+3αHP (inset). The experiment was terminated on day 63. Values in brackets denote number of mice, out of six, with tumors for each treatment group. Note that palpable tumors were first detected on day 50 in controls, and on days 40, 55 and 46 in the 5αP, 3αHP and 5αP+3αHP treated mice, respectively. Data points represent size (mm^3; mean + SEM) *$P < 0.05$ compared to control.
